# Theories and Practices in Learning and Assessment for Postgraduate Medical Education: A Review

**DOI:** 10.7759/cureus.74160

**Published:** 2024-11-21

**Authors:** Omar M Ismail, Umar N Said, Omar El-Omar, Mohammed A Bhutta

**Affiliations:** 1 Department of Trauma and Orthopaedics, Northern Care Alliance NHS Foundation Trust, Manchester, GBR; 2 Trauma and Orthopaedics, University Hospitals Coventry and Warwickshire NHS Trust, Coventry, GBR; 3 Department of Trauma and Orthopaedics, Calderdale and Huddersfield NHS Foundation Trust, Huddersfield, GBR

**Keywords:** assessment strategies, clinical competence, formative assessments, learning theories, maslow’s hierarchy in education, medical education, medical education feedback models, miller’s pyramid, postgraduate medical education, summative assessments

## Abstract

This literature review explores key theories and practical strategies in postgraduate medical education. It examines essential learning strategies, such as didactic and experiential teaching methods, structured lesson planning, and models such as Maslow’s hierarchy and Kolb’s experiential learning cycle. Active learning techniques and feedback models, crucial for guiding medical trainees’ growth, are also discussed. The review then shifts focus to assessment, looking at both formative and summative approaches, Miller’s pyramid of competence, and Van der Vleuten’s utility equation. By evaluating assessment formats, blueprinting, and feedback, this review offers insights into educational strategies that enhance postgraduate medical training.

## Introduction and background

In postgraduate medical education, effective teaching and assessment strategies are essential for developing a high standard of clinical skills and knowledge in trainee doctors. Trainees must not only gain medical knowledge but also utilise it in diverse and unpredictable situations due to the increasing complexity of modern healthcare. Therefore, it is the responsibility of medical teachers to move past basic knowledge delivery, encourage analytical thinking and practical skill utilisation, and enhance memory retention, which are essential for doctors making clinical decisions and providing patient care.

To this end, assessment becomes a vital tool for measuring learner competency, offering structured feedback, and providing insights that enhance curriculum effectiveness. A well-rounded assessment approach also allows educators to make informed judgments about a trainee's progress and readiness to advance through the training stages. This literature review examines foundational theories and practical applications in postgraduate medical education, exploring both the teaching strategies that support clinical skills development and the assessment techniques that validate competency and facilitate feedback for continuous improvement. This review adds a concise overview of these subjects to the existing literature, which would be of benefit to educational leaders within postgraduate medical education for course delivery.

To retrieve the literature on this topic, using the Ovid platform, keywords such as “pedagogy”, “medical training” and “assessment” filtered results. The database used was MEDLINE. Mainly, literature published after 1980 was included (other than pertinent crucial literature prior to this to ensure a complete review) and was ranked using the Centre for Reviews and Dissemination standards.

## Review

Learning theories and strategies in medical education

Didactic Teaching and Lesson Planning

Didactic teaching, such as lectures, remains a cornerstone in medical education, offering a structured way to deliver foundational information efficiently. When well-executed, lectures not only benefit learners but also reinforce the presenter’s own knowledge, as teaching requires careful preparation and synthesis of material [1]. While lectures can efficiently cover substantial amounts of information, the impact of a lecture and retention of information depends heavily on how it is structured and delivered.

Effective lesson planning is essential in didactic teaching, particularly for postgraduate learners who bring varying degrees of prior experience to each session [2]. Van Diggele suggested that a well-structured lesson plan should include an assessment of the audience’s background and resources, clearly defined learning outcomes, engaging activities with appropriate resources, assessments aligned with learning needs and a concise summary of key take-home messages [3]. A thoroughly developed lesson plan connects all parts of the session to enhance learning and memory, guaranteeing that the instruction meets the specific needs of postgraduate students.

Online Learning: Opportunities and Challenges

After the onset of the COVID-19 pandemic, with advancements in digital technology, online learning has become more prevalent in medical education. Its flexibility allows trainees to attend sessions from various locations and often at times that best fit their schedules. However, challenges unique to online learning persist, such as connectivity issues, lack of a conducive learning environment, and self-discipline hurdles, all of which can compromise engagement and effectiveness [4,5]. These limitations make traditional, in-person training a preferred approach in clinical settings, as it enables hands-on practice and immediate feedback that are difficult to replicate online.

When online learning is incorporated into a curriculum or teaching programme, careful planning becomes crucial to maintain engagement and interactivity. Effective strategies may include the use of multimedia content, interactive case studies, and discussion forums to encourage participation and ensure that learning outcomes align closely with clinical competencies.

Supportive Learning Environments: Maslow’s Hierarchy of Needs

**Figure 1:**
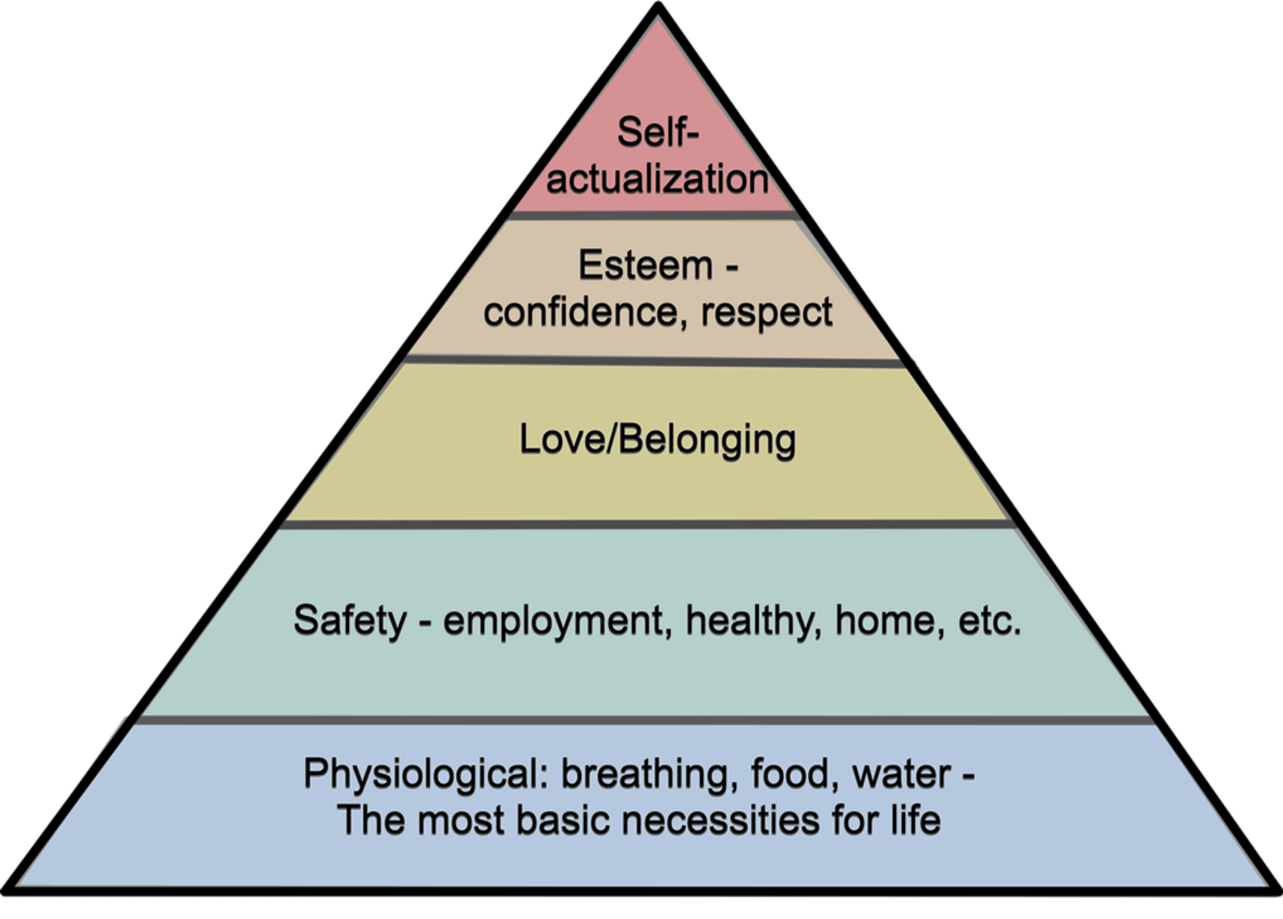
Maslow’s hierarchy of needs Taken from [7], with permission

A conducive learning environment is fundamental to the success of any educational approach. Maslow’s Hierarchy of Needs offers a practical framework for designing such environments, beginning with meeting basic needs like physical comfort and accessibility. In the context of medical education, this can include providing comfortable seating, necessary equipment, and central locations that reduce logistical stress for learners, particularly those in demanding medical rotations that try to fit teaching in while ensuring they complete their allocated workload [6]. Paying heed to these fundamental needs can establish a nurturing environment that enables learners to concentrate on their education instead of their physical discomfort or logistical obstacles (Figure 1).

Maslow’s theory also highlights psychological needs, such as feelings of belonging and respect, which can be fulfilled through the promotion of a culture of learning that is supportive and respectful [6]. This culture encourages participation and teamwork, making learners feel appreciated and heard by both peers and instructors, improving their educational journey.

Experiential Learning: Kolb’s Model

**Figure 2:**
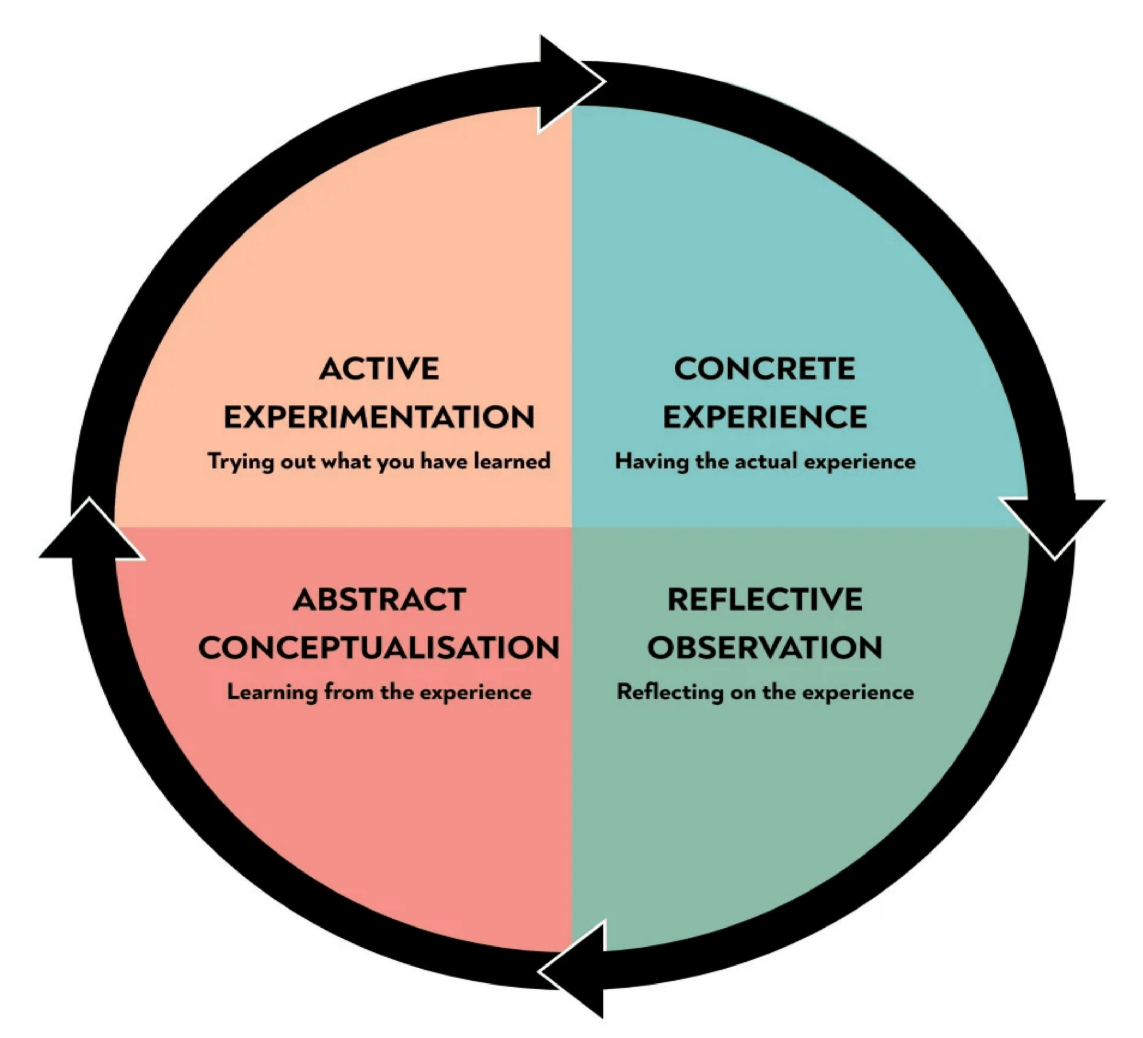
Kolb's experiential learning cycle Taken from [9], with permission

Kolb’s experiential learning model is also highly applicable to postgraduate medical education due to its emphasis on reflective practice and learning through experience [8]. The model is structured as a continuous cycle: concrete experience, reflective observation, abstract conceptualisation, and active experimentation. This approach allows learners to internalise theoretical knowledge by applying it in clinical settings, reflecting on these experiences, and gradually improving through feedback and repetition [8]. This experiential cycle is not unlike the famous adage “see one, do one, teach one” commonly cited in medical training (Figure 2).

By using Kolb’s model, educators can guide learners through clinical experiences, helping them develop critical thinking skills and adapt their approaches to different clinical scenarios. This reflection and application process not only builds competence but also fosters a sense of professional responsibility as trainees recognize the real-world impact of their clinical decisions.

Visual Aids and Active Learning Techniques

Visual aids have been shown to significantly enhance learning, especially in fields like medicine, where complex information often needs to be clarified for better comprehension. Studies indicate that visual explanations improve information retention, making them particularly valuable for explaining medical procedures and physiological processes [10].

Additionally, active learning techniques, such as small group teaching, promote engagement and deeper understanding through discussion, peer interaction, and immediate feedback [11]. Biggs emphasises the importance of active learning in achieving meaningful, long-term retention, highlighting that a structured knowledge base and motivating context are essential to successful learning [12].

Feedback models

Feedback is a vital component of effective learning in medical education, guiding trainees toward continuous improvement. Pendleton’s feedback model provides a structured and supportive approach to feedback that encourages learners to reflect on their performance and identify areas for growth [13]. Instructors first ask learners to discuss what went well, then offer additional insights on areas for improvement, and finally allow learners to set goals for future practice [13].

An alternative model, often referred to as the “feedback sandwich,” combines constructive criticism with positive reinforcement [14]. This approach maintains motivation and helps prevent learners from feeling discouraged, making it easier for them to focus on the actionable aspects of feedback [14].

Assessment approaches and theoretical models

Assessments are carried out to examine the effectiveness of teaching and gain insight into the performance and aptitude of the student. Erwin stated that assessment is “the process of defining, selecting, designing, collecting, analysing, interpreting, and using information to increase students’ learning and development” [15]. Assessments are usually performed to achieve one or more of the following: provide certification for a qualification of the course content, improve student learning of the course and contribute to quality assurance to ensure that the course and its content are appropriate and held to a rigorous standard [16,17].

Assessments can be divided into formative and summative assessments as described by Wass [18]. Formative assessments, such as workplace-based assessments (WBPAs), provide ongoing feedback that enables instructors to track performance, identify areas for improvement and support learners in meeting clinical standards. These assessments are designed to provide formative insights without penalising trainees, thereby encouraging continuous learning and improvement.

Summative assessments, such as speciality training exit exams, evaluate learners’ competencies at key transition points in their education. These high-stakes evaluations determine whether learners are prepared for independent practice or advancement to more specialised training, often forming a critical component of postgraduate medical education.

Miller’s Pyramid, the Utility Equation and Blueprinting

**Figure 3:**
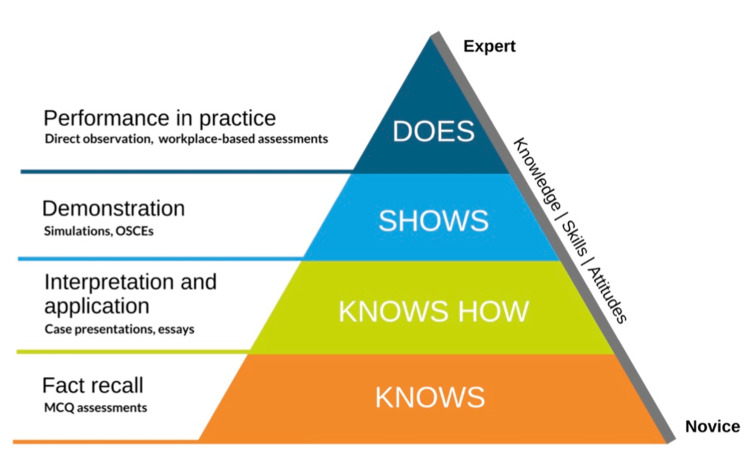
Miller's pyramid demonstrating the hierarchical steps of increasing competence Taken from [21], with permission

Miller’s pyramid offers a widely adopted framework for assessing clinical competence, organising learning outcomes from foundational knowledge ("knows") to practical skill application ("does") (Figure 3) [19,20]. This hierarchical model provides a structured way to design assessments that evaluate different levels of learning such as multiple-choice questions (MCQs) for knowledge and objective structured clinical examinations (OSCEs) for practical skills.

The utility equation described by Van der Vleuten is a combination of five factors - reliability, validity, feasibility, acceptability, and educational impact - to ensure an assessment is reproducible, accurately measures its intended outcomes, is cost-effective, is acceptable to all stakeholders and positively influences learner behaviour [22]. The ideal assessment should encompass all these values to achieve a high utility. Assessments can never be perfect, and the key is to strike a balance between the different components through compromise and the practicalities of the assessment. For that reason, this utility equation should not just be applied to one form of assessment but to the overall process. For example, a summative examination can involve an MCQ (assessing the cognition steps of Miller’s pyramid) and an OSCE (assessing the behaviour of Miller’s pyramid). The utility equation should therefore be applied to the whole process to ensure it is a summative examination.

Van der Vleuten suggested that a variety of assessment formats help improve the reliability and validity of the results [22]. Different formats of assessments fulfil the different aspects of the utility equation, so in tandem, two different formats would have a higher utility than the separate assessments. Medical school or higher medical training examinations would have an MCQ component (assessing the cognition steps of Miller’s pyramid) and an OSCE or WBPA component (assessing the behaviour steps of Miller’s pyramid) [19].

Blueprinting further ensures alignment between assessments and curriculum objectives. A test blueprint refers to the key concepts of an assessment, the content that needs to be tested, the appropriate amount of weighting per topic, deciding the number of questions per topic, and informing the faculty and learners of the blueprint in advance [23,24]. Blueprinting ensures that the assessments are valid by ensuring the learning objectives of the programme match those of the assessment and helps provide a structure for the examination [25].

Assessment Methods Used in Postgraduate Medical Education

Various assessment formats offer distinct advantages and limitations. MCQs are the most used question type, as they are relatively easy to construct and conduct (high feasibility) and have high reliability as they are objective. They can be re-used for future cohorts and there is evidence of a strong correlation of scores between MCQs and free-text answers [26]. However, they can have low validity if used as the sole assessment, and there is a potential for ambiguity with the questions and no chance to elaborate. MCQs also mainly test factual recall rather than higher steps of cognition on Miller’s pyramid [19,26].

WBPA assessments can be conducted contemporaneously during shifts with real patients with debriefing afterwards. A senior would sit with the medical trainee and ask them questions about a case. Questions asked should allow learners to speak without interruption and be given time to think. They should also only answer one question at a time. This improves student satisfaction and participation and can lead to higher-quality responses [27].

WBPAs are scored on a scale where each level is mapped to a certain level of competency. However, WBPAs and OSCEs have an inherent subjectivity among different supervisors of varying strictness (which can be compensated for by quality assurance). Other disadvantages are that WBPAs can be more time-intensive and require more resources and staff as compared to an MCQ (thus more expensive) [28]. Some students can struggle with high levels of stress during OSCEs that can hamper performance and may adversely affect the validity and reliability of OSCEs [29]. However, these assessments have been shown to improve clinical skills [30], allow for interaction between the student and examiner and provide an opportunity for self-learning [31], and the results are representative of the quality of teaching [32].

Feedback After Assessments

After these assessments, it is necessary to provide feedback to the candidate, and for the candidate to provide feedback on the assessments to ensure that they are acceptable. Wiggins suggested giving feedback should be goal-referenced, tangible, actionable, comprehensible, timely, continuous over future interactions relating to the teaching session, and consistent in quality among all supervisors [33]. Oral feedback can be given immediately, and written feedback can be provided later so the student can use it as a reference.

## Conclusions

In postgraduate medical education, effective teaching and assessment strategies are essential for developing skilled and knowledgeable medical professionals. Theories such as Maslow’s hierarchy and Kolb’s experiential learning model influence the development of supportive environments while structured lesson planning, active learning techniques, and feedback models provide crucial support for learner growth.

In addition, formative and summative assessments validate skills and offer feedback necessary for the development of clinical competencies. Frameworks like Miller’s pyramid and Van der Vleuten’s utility equation guide assessment design while blueprinting ensures assessments align with curriculum objectives. Together, these strategies provide a foundation for training medical professionals who are prepared for the demands of clinical practice.
